# Successful Treatment of Infectious Endocarditis Associated Glomerulonephritis Mimicking C3 Glomerulonephritis in a Case with No Previous Cardiac Disease

**DOI:** 10.1155/2014/569047

**Published:** 2014-11-23

**Authors:** Yosuke Kawamorita, Yoshihide Fujigaki, Atsuko Imase, Shigeyuki Arai, Yoshifuru Tamura, Masayuki Tanemoto, Hiroshi Uozaki, Yutaka Yamaguchi, Shunya Uchida

**Affiliations:** ^1^Department of Internal Medicine, Teikyo University School of Medicine, 2-11-1 Kaga, Itabashi-ku, Tokyo 173-8605, Japan; ^2^Department of Pathology, Teikyo University School of Medicine, 2-11-1 Kaga, Itabashi-ku, Tokyo 173-8605, Japan; ^3^Yamaguchi's Pathology Laboratory, 20-31-1 Minoridai, Matsudo-shi, Chiba 270-2231, Japan

## Abstract

We report a 42-year-old man with subacute infectious endocarditis (IE) with septic pulmonary embolism, presenting rapidly progressive glomerulonephritis and positive proteinase 3-anti-neutrophil cytoplasmic antibody (PR3-ANCA). He had no previous history of heart disease. Renal histology revealed diffuse endocapillary proliferative glomerulonephritis with complement 3- (C3-) dominant staining and subendothelial electron dense deposit, mimicking C3 glomerulonephritis. Successful treatment of IE with valve plastic surgery gradually ameliorated hypocomplementemia and renal failure; thus C3 glomerulonephritis-like lesion in this case was classified as postinfectious glomerulonephritis. IE associated glomerulonephritis is relatively rare, especially in cases with no previous history of valvular disease of the heart like our case. This case also reemphasizes the broad differential diagnosis of renal involvement in IE.

## 1. Introduction

Infectious endocarditis (IE) has various renal histologies, including renal infarction due to septic emboli, acute postinfectious glomerulonephritis, membranoproliferative glomerulonephritis, immune or pauci-immune crescentic glomerulonephritis, and acute interstitial nephritis [1–3]. Some patients with IE show vasculitis-like general symptoms, renal failure, and positive PR3-ANCA with or without ANCA-related vasculitis. Thus, it is important to evaluate renal histopathology in patients with IE-related renal involvement to determine whether immunosuppressive therapy should be introduced.

The term C3 glomerulopathy is recently defined by the pathological findings of complement 3 (C3) which is deposited within the glomerulus in the absence of substantial immunoglobulin, and there may remain much room for discussion. Dysregulation of the alternative pathway of complement is brought by genetic and/or acquired defects, with interindividual variability giving rise to two broad subtypes of C3 glomerulopathy-membranoproliferative glomerulonephritis (dense deposit disease) and C3 glomerulonephritis [4]. It is known that C3 glomerulopathy may present following an infectious episode. It is not uncommon that typical cases of postinfectious glomerulonephritis show deposition of C3 without immunoglobulin [5]. In these cases, distinction of C3 glomerulopathy will depend on the absence of atypical features on light microscopy and electron microscopy and also on a typical clinical course with resolution [6].

Here we describe a case with no previous history of heart disease, presenting subacute IE associated with septic pulmonary embolism, rapidly progressive glomerulonephritis, and positive PR3-ANCA. Differential diagnosis by renal histopathology could not initially rule out C3 glomerulonephritis, but the case was classified as postinfectious glomerulonephritis because the complement and renal dysfunction were recovered after treatment of IE.

## 2. Case Presentation

A 42-year-old Japanese man was referred to our hospital because of fever, appetite loss, myalgia, pain, and swelling of the left foot, leg edema, decreased renal function, and multiple nodules and cavities in bilateral lungs on computed tomography (CT) scan. The patient had been well until 6 weeks earlier, when he went to orthopedics about lumbago and dry cough. He consulted a doctor about leg edema 5 days ago and appetite loss and pain and swelling on the left foot 2 weeks ago. He was referred to local hospital and then transferred to our hospital on the same day because his laboratory data showed Hb of 8.8 g/dL, serum creatinine (Cr) of 3.87 mg/dL, and C-reactive protein of 32.0 mg/dL and multiple pulmonary nodules and cavities on CT scan.

Physical examination on admission showed the temperature 38.2°C, the blood pressure 106/64 mm Hg, the pulse 112 beats per minute, the respiratory rate 26 breaths per minute, and the oxygen saturation 100%. He had dry tongue, pain, redness, and swelling at the left dorsum of the foot and pitting edema on both legs. He had no symptom or sign of vasculitis on eyes, ears, and upper respiratory tract.

Laboratory-test results are shown in the Table 1, indicating anemia, hypoalbuminemia, renal failure with proteinuria and pyuria, and positive inflammatory signs. Saline and ceftriaxone (CTRX) were infused intravenously for dehydration and cellulitis at the left dorsum of the foot, respectively.

**Figure 1:**
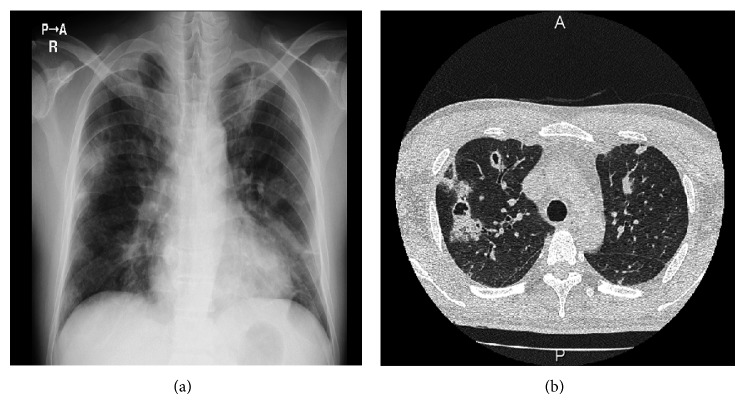
(a) Chest X-ray showing multiple bilateral nodular densities. (b) CT of the chest demonstrating bilateral multiple lung nodules, some of which are cavitated.

**Figure 2:**
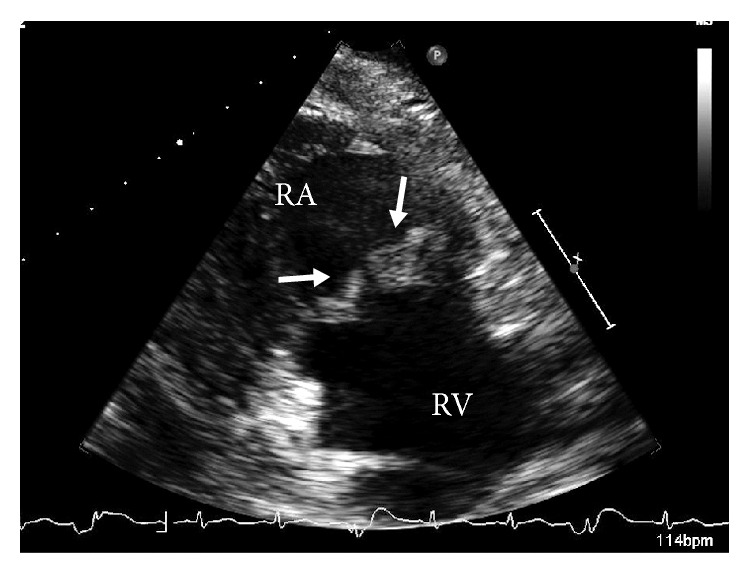
Transthoracic echocardiograph. Extensive bacterial vegetations are observed on the tricuspid (arrows) valves. RA: right atrium, RV: right ventricle.

3 days after hydration, serum Cr level was decreased from 6.11 mg/dL to 3.69 mg/dL but was sustained around 2 to 3 mg/dL. Urinalysis began to show significant hematuria. PR3-ANCA was revealed to be positive. Thus, the findings of general symptom, rapidly progressive glomerulonephritis, positive PR3-ANCA, nodules on chest X-ray (Figure 1(a)), and multiple cavities on chest CT scan (Figure 1(b)), raised suspicion of granulomatosis with polyangiitis with renal involvement. Although the patient had no previous history of cardiac disease, echo cardiography showed tricuspid valve regurgitation with vegetation 17 mm × 11 mm in diameter (Figure 2). Blood culture revealed positive methicillin-susceptible* Staphylococcus aureus*; thus the patient was diagnosed to be subacute IE. Cellulitis may be a cause of IE in this case. Pulmonary multiple cavities should be caused by septic pulmonary embolism. Methicillin-susceptible* Staphylococcus aureus* was sensitive to cefmetazole (CEZ) and CEZ was started as substitute for CTRX.

**Figure 3:**
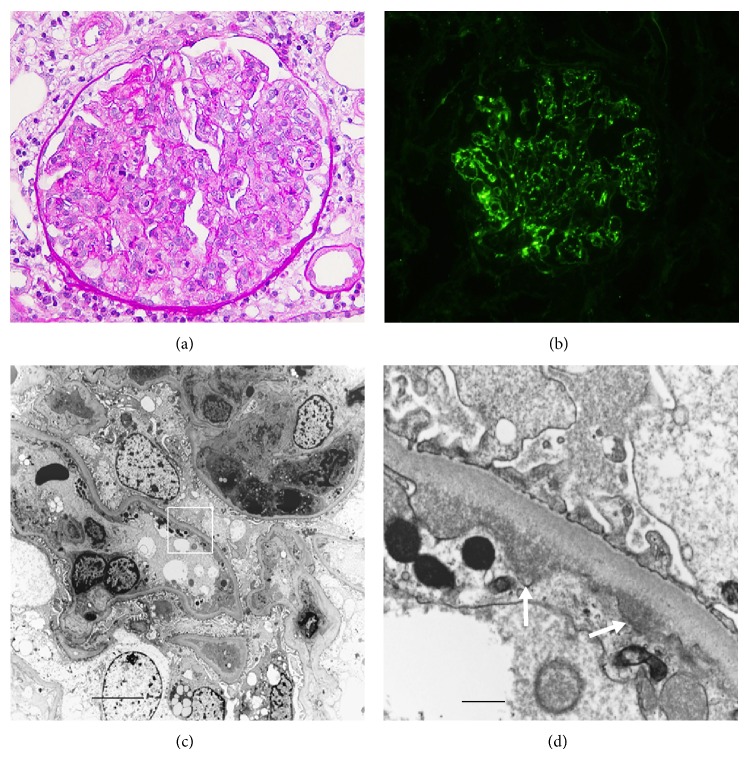
Photomicrographs of renal tissue. (a) Light microscopy shows a diffuse endocapillary proliferative glomerulonephritis with lobular formation. PAS staining. Original magnification ×400. (b) Bright C3 staining along capillary walls by immunofluorescence. (c) Electron microscopy shows polymorphonuclear cells and monocyte infiltration in the capillary wall. Bar = 2.0 *µ*m. (d) The area of the square in Figure 3(c) shows subendothelial deposits (arrows). Bar = 0.2 *µ*m.

Renal biopsy 2 weeks after CEZ treatment showed no global sclerosis out of 12 obtained glomeruli. Each glomerulus revealed no crescent formation but diffuse endocapillary proliferative glomerulonephritis (Figure 3(a)) with starry sky pattern of significant C3 deposition (Figure 3(b)) and minimal to absent immunoglobulins by immunofluorescence. Electron microscopy showed polymorphonuclear cells and monocytes infiltration in glomerular capillaries (Figure 3(c)) and the electron dense deposits in the subendothelial areas but no hump formation (Figure 3(d)). Our patient had IE associated with septic pulmonary embolism, and it is reported that some of glomerulonephritis associated with IE and glomerulonephritis associated with visceral infection also show the glomerular lesions with dominant C3 deposition [7] similar to that in our case. Glomerulonephritis with dominant C3 deposition in our patient could not rule out C3 glomerulonephritis at the time of renal biopsy based on C3 glomerulopathy: consensus report [6]. Renal infarction and abscess were not found in the renal specimens and were not suggested by echo and CT scan.

**Figure 4:**
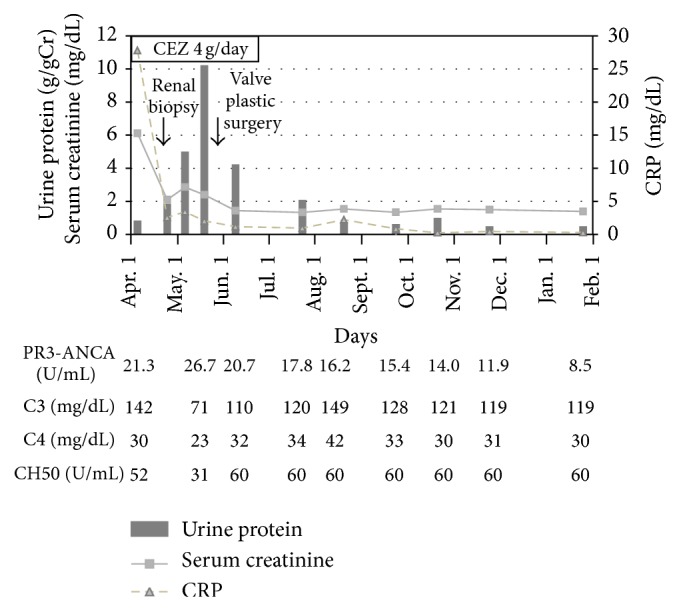
Clinical course after admission.

Clinical course after admission was shown in Figure 4. C3 was found to be decreased at the time of renal biopsy. Although treatment with CEZ 4 g/day brought negative blood culture and partially ameliorated renal function, proteinuria began to increase and peaked 10 g/gCr 6 weeks after CEZ administration. Since vegetation grew to 27 mm × 15 mm in diameter at tricuspid valve, tricuspid valve plastic surgery was performed. Additional 6 weeks of treatment with CEZ after the operation got IE cured. Successful treatment of IE ameliorated hypocomplementemia and renal failure with active urinalysis. The titer of PR3-ANCA was gradually decreased and nodular lesions on chest X-ray disappeared after recovery from subacute IE. Ten months after the operation, the patient is well with proteinuria of 0.16 g/gCr, Cr of 1.32 mg/dL, C3 of 115 mg/dL, and PR3-ANCA of 8.9 U/mL.

## 3. Discussion

It is reported that around 75% of patients with IE have an underlying structural heart disease at the time when IE is diagnosed [8]. Glomerulonephritis associated with IE shows various renal lesions and is relatively rare, especially in cases with no previous heart disease like our case. ANCA, including PR3- and MPO-ANCA, occurs in a substantial proportion of IE cases with or without renal vasculitis. It is reported that, among 109 IE cases, 18% had cytoplasmic and/or perinuclear ANCA and 8% had PR3-ANCA or MPO-ANCA [9]. IE cases could present clinical manifestations that mimic a primary systemic vasculitis. Among IE cases with ANCA-positivity, some cases may develop vasculitis with crescentic glomerulonephritis [3, 9]. Thus, differential diagnosis of renal involvement of IE is important to make a decision on whether immunosuppressive therapy should be introduced. Our case did not show ANCA-associated glomerulonephritis. The PR3-ANCA in this case seemed to be just the epiphenomenon of the systemic infection.

Our case revealed diffuse endocapillary glomerulonephritis with C3-dominant immunofluorescence staining with minimal immunoglobulins. There were only subendothelial electron dense deposits without hump formation. It is rare, but a similar electron microscopic finding of glomerular lesion was reported in some cases with acute glomerulonephritis caused by staphylococcal [10] or streptococcal [11] infection. It is reported that glomerulonephritis associated with IE and glomerulonephritis associated with visceral infection especially secondary to staphylococcal infection include the glomerular lesions with dominant C3 deposition [7]. Sethi et al. reported that there were patients who show an underlying abnormality of the alternative pathway of complement among patients with persistent hematuria and proteinuria even after resolution of the infection, diagnosed with postinfectious glomerulonephritis [5]. They called them “atypical” postinfectious glomerulonephritis, showing the diffuse (endocapillary) proliferative pattern of glomerulonephritis and the possible presence of immunoglobulins and numerous subepithelial humps with mesangial and subendothelial deposits. They speculated that there might be an infectious trigger, which is the underlying abnormality of the alternative pathway that drives the disease process. It is known that C3 glomerulopathy often shows membranoproliferative glomerulonephritis but also several other renal histologies [12]. Some C3 glomerulonephritis was reported to be masqueraded as acute postinfectious glomerulonephritis [13]. Therefore, C3 glomerulonephritis was not pathologically excluded in our patient [6]. However, successful treatment of IE ameliorated renal failure with active urinalysis and hypocomplementemia. Thus, renal pathology of dominant C3 deposition in our case was classified as postinfectious glomerulonephritis [6].

Our case showed normocomplementemia on admission but exhibited transient low grade hypocomplementemia with decreased C3 during active IE. As several complement proteins, including C3 and C4, behave as acute phase proteins, an increased synthesis may mask accelerated catabolism. Therefore, the assessment of complement protein levels is often inadequate to detect complement activation. In addition, the range of normal complement protein concentrations is wide, thus low levels may be related to deficiency state [14].

PR-3 ANCA associated with IE was reported to be positive for several months to years after IE was cured [1, 15]. In accordance with the report, our case sustained low titer PR3-ANCA 3 to 4 months after IE was cured (stopping antibiotics) and then PR3-ANCA was declined in one half.

In summary, IE associated glomerulonephritis is relatively rare, especially in cases with no previous history of heart disease like our case. Successful treatment of IE ameliorated hypocomplementemia and renal failure; thus C3 glomerulonephritis-like lesion in our case was classified as postinfectious glomerulonephritis. The present case highlights the broad differential diagnosis of renal involvement in IE, including C3 glomerulonephritis triggered by IE.

## Figures and Tables

**Table 1:** Laboratory data at the time of admission.

Blood		Urine	
Blood count		Dipstick test	
White blood cell	13,400/mm^3^	Protein	1+
Eosinophil	0%	Glucose	(−)
Red blood cell	295 × 10^4^/mm^3^	Occult blood	(±)
Hemoglobin	8.7 g/dL	Sediment	
Hematocrit	24.7%	Leukocyte	10–19/HPF
Platelet	5.0 × 10^4^/mm^3^	Red blood cell	1–4/HPF
Biochemical tests		Epithelial cell	0-1/HPF
Total protein	6.4 g/dL	Red blood cell cast	0-1/HPF
Albumin	1.5 g/dL	Biochemical analysis	
Aspartate-aminotransferase	24 IU	Urine protein	0.61 g/gCr
Alanine-aminotransferase	22 IU	N-Acetyl-*β*-D-glucosaminidase	52.3 U/mL
Lactate dehydrogenase	220 IU	*β*2-Microglobulin	3,720 *μ*g/L
Blood urea nitrogen	97.5 mg/dL	*α*1-Microglobulin	180 mg/L
Cr	6.11 mg/dL		
Uric acid	14.5 mg/dL		
Sodium	134 mEq/L		
Potassium	4.5 mEq/L		
Chloride	94 mEq/L		
Calcium	7.6 mg/dL		
Phosphate	5.5 mg/dL		
Creatine kinase	43 IU/L		
Fasting blood glucose	111 mg/dL		
HbA1c	5.8%		
eGFR	9.2 mL/min/1.73 m^2^		
Immunology			
C-reactive protein	27.84 mg/dL		
Procalcitonin	4.0 ng/mL		
Immunoglobulin G	2,190 mg/dL		
Immunoglobulin A	364 mg/dL		
Immunoglobulin M	87 mg/dL		
Complement 3	142 mg/dL (65–135)		
Complement 4	30 mg/dL (13–35)		
CH50	52 U/mL (22–58)		
Anti-streptolysin O	37.6 U/mL		
Hepatitis B surface antigen	(−)		
Hepatitis C virus antibody	(−)		
Human immunodeficiency virus antibody	(−)		
RF	10.0 U/mL		
Antinuclear antibody	(−)		
MPO-ANCA	1.0 U/mL (<3.5)		
PR3-ANCA	21.3 U/mL (<3.5)		
Cryoglobulin	(−)		

Creatinine: Cr; MPO-ANCA: myeloperoxidase-anti-neutrophil cytoplasmic antibody; PR3-ANCA: proteinase 3-anti-neutrophil cytoplasmic antibody. The figure in the parenthesis shows the normal range.
